# Remote Technology-Based Training Programs for Children with Acquired Brain Injury: A Systematic Review and a Meta-Analytic Exploration

**DOI:** 10.1155/2019/1346987

**Published:** 2019-08-01

**Authors:** Claudia Corti, Viola Oldrati, Maria Chiara Oprandi, Elisabetta Ferrari, Geraldina Poggi, Renato Borgatti, Cosimo Urgesi, Alessandra Bardoni

**Affiliations:** ^1^Scientific Institute, IRCCS E. Medea, Bosisio Parini, Lecco, Italy; ^2^Laboratory of Cognitive Neuroscience, Department of Languages and Literatures, Communication, Education and Society, University of Udine, Italy; ^3^Scientific Institute, IRCCS E. Medea, San Vito al Tagliamento, Pordenone, Italy

## Abstract

**Introduction:**

Multidisciplinary rehabilitation interventions are considered to be a need for children with acquired brain injury (ABI), in order to remediate the important sequelae and promote adjustment. Technology-based treatments represent a promising field inside the rehabilitation area, as they allow delivering interventions in ecological settings and creating amusing exercises that may favor engagement. In this work, we present an overview of remote technology-based training programs (TP) addressing cognitive and behavioral issues delivered to children with ABI and complement it with the results of a meta-analytic exploration.

**Evidence Acquisition:**

We performed the review process between January and February 2019. 32 studies were included in the review, of which 14 were further selected to be included in the meta-analysis on TP efficacy.

**Evidence Synthesis:**

Based on the review process, the majority of TP addressing cognitive issues and all TP focusing on behavioral issues were found to be effective. Two meta-analytic models examining the means of either cognitive TP outcomes or behavioral TP outcomes as input outcome yielded a nonsignificant effect size for cognitive TP and a low-moderate effect size for behavioral TP. Additional models on outcomes reflecting the greatest beneficial effects of TP yielded significant moderate effect sizes for both types of TP. Nevertheless, consistent methodological heterogeneity was observed, pointing to cautious interpretation of findings. A subgroup analysis on visuospatial skill outcomes showed a smaller yet significant effect size of cognitive TP, with low heterogeneity, providing a more reliable estimation of overall cognitive TP effects.

**Conclusions:**

Promising results on remote cognitive and behavioral TP efficacy emerged both at the review process and at the meta-analytic investigation. Nevertheless, the high heterogeneity that emerged across studies prevents us from drawing definite conclusions. Further research is needed to identify whether specific training characteristics and population subgroups are more likely to be associated with greater training efficacy.

## 1. Introduction

Pediatric acquired brain injuries (ABI) are insults to the central nervous system that occur after birth. The nature of this damage can be traumatic or nontraumatic (infection, stroke, hypoxia, brain tumors, neurosurgery, and other central nervous system treatments [1]). Traumatic brain injury (TBI) is the most frequent cause of ABI in childhood [2], affecting more than 3 million children worldwide every year [3] and representing a considerable portion of all neurologic disabilities [4].

Children with ABI are often reported to exhibit deficits in various cognitive domains, including intellectual functioning, core cognitive abilities, and visual-related processes [2, 5–7]. Even those children who keep an intellectual quotient within the normal range often experience difficulties in achieving academic success after the event [8, 9]. Deficits in neuropsychological abilities affecting social competence and psychosocial and behavioral concerns have also been documented [10, 11]. Anomalies in cognitive, behavioral, and social domains have been reported regardless of injury severity, thus affecting the majority of children [12]. All these deficits cause a negative impact on children's adjustment in daily life [13]. Consequently, rehabilitation treatments to improve cognitive and behavioral functioning have been considered necessary for sustaining recovery and adjustment of these patients [12, 14].

In the last two decades, numerous papers on rehabilitation treatments for pediatric ABI or reviews on the topic have been published [1, 12, 14–20]. However, it has not been yet clarified which are the most effective interventions. This could be associated with methodological shortcomings of the study design, such as lack of control group or small sample size, or to the heterogeneity of the population selected for the study (in terms of age at the event, time since the event, diagnosis, etc.), or even to the variety of instruments used to assess outcomes [12, 17].

A review of Lindsay and colleagues [16] described hospital-to-school reintegration interventions for pediatric ABI, including treatments addressing cognitive or behavioral impairments, interventions delivered by a clinician in the clinical setting, home-based or web-based interventions, family or social support programs, and art-based activities. The Authors identified some possible components of successful interventions, such as one-to-one sessions conducted by a therapist or educator, the involvement of parents, and homework activities. However, they also reported that a significant improvement could be observed after online home-based interventions addressing cognitive and behavioral aspects [21–23]. This pointed to positive gains associated with computer-based treatments performed out of the clinical setting and with limited participation of a therapist.

Linden and colleagues [14, 15] performed two meta-analyses on web-based training programs (TP) for pediatric ABI. The meta-analysis focusing on neuropsychological outcomes [15] was based on three studies from the same research team applying web-based interventions for everyday executive functions and found positive treatment effects on self- and/or parent-report measures on everyday behavioral problems associated with executive dysfunction. However, potential sources of methodological biases were reported. On the contrary, the meta-analysis focusing on academic achievement [14] found no training effect. Nevertheless, the small number of participants in the included studies and the variety of measures adopted to assess outcomes were indicated as potential factors limiting the validity of conclusions.

A recent review of Resch and colleagues [17] provided an overview of the studies that described cognitive rehabilitation interventions for ABI in pediatric age, examining their specific components and distinguishing them as (1) metacognition and/or strategy use; (2) computerized drill-based exercises; (3) interventions combining metacognition and/or strategy use and drill-based exercises; and (4) external aids. Overall, this work indicated that metacognition and/or strategy-use interventions may produce benefits for adaptive behavior but have limited effects for cognitive functions. In contrast, computerized drill-based exercises seem to generate only near-transfer gains, namely in those outcomes that are similar to the training tasks, while far transfer does not emerge either in the global cognitive domain or in more general executive-function behavior or adaptive behavior. The Authors suggested that such results could be in part attributed to short training duration and short training time. The simultaneous adoption of both metacognition/strategy-use and computerized drill-based training was identified as the most effective way to improve cognitive and psychosocial impairments. External aids were reported to generate benefits in everyday cognitive functions, particularly when they are used in combination with metacognition/strategy use. Finally, the provision of the interventions in family- or peer-supported settings was indicated to be a potential factor leading to better outcomes.

In a similar vein, Wade and colleagues [12] described technology-assisted interventions for pediatric patients with ABI, focusing on both treatments delivered at a distance and treatments included in the traditional rehabilitation setting to promote engagement and improve efficacy. Overall, the most positive results were reported for those interventions providing family-centered, online problem-solving aimed at ameliorating behavioral issues. In three subsequent meta-analytic explorations from the same research group, these interventions were found to be more effective when being delivered at longer time since injury and to children of older age (in front of an age range of 5-18 years), with lower intellectual quotient and whose parents had lower education [24–26]. Treatments for attention, working memory, and executive functioning were also described as promising, but methodological limitations of studies were highlighted. Furthermore, commercially available games were indicated as alternatives to support physical and occupational therapy, while training in the use of social media and app-based goal setting were described as having the potential advantage to promote social engagement.

Taken together, the cited reviews indicated the use of technology as a promising method to implement rehabilitation interventions for children with ABI, even though they warned about the fact that definite conclusions are hampered by methodological issues, such as heterogeneity in patients and outcomes and lack of control groups. These reviews included a complete and updated overview of the treatments for ABI but did not distinguish between treatments delivered in a clinical setting and those delivered remotely. The use of remote interventions has consistently increased in recent years [27, 28] with the aim to overcome the limits associated with traditional interventions, such as high time demand, elevated costs, accessibility issues, and heterogeneity in treatment practices. Remote TP may increase opportunities and consistency of rehabilitation for patients, limiting time and economic demands for them and their families, and may also promote engagement, introducing amusing exercises by using computer-based platforms [12, 29]. In view of the peculiar characteristics of remote TP, evaluating the effects of these interventions, distinguishing them from those delivered in a traditional clinical setting, should be considered a matter of interest. For this reason, different from the reviews described above, the present review focused on the effects of TP for pediatric ABI that were delivered in an ecological setting only and that did not require the intervention of a therapist at all or that required it only to a limited extent (at most, once a week).

Remote TP with either a cognitive or a behavioral focus were considered for this work. However, due to the methodological differences between the two types of interventions, cognitive and behavioral TP were examined separately. In fact, cognitive TP were composed of drill-based exercises aimed at stimulating different cognitive domains and required solely the involvement of patients. In most studies, the therapist intervention was limited to monitor training adherence, while in a few studies it was aimed at providing weekly feedback on patient's performance and cognitive strategies. In contrast, behavioral TP consisted of self-guided didactic content on problem-solving abilities and on exercises to practice these skills and were performed by the patient together with family or with the remote support of a peer. Moreover, for most behavioral TP, every one to two weeks a therapist performed a remote videoconference session with the patients and families to give a feedback on the implementation of the problem-solving process in real life. Nevertheless, despite methodological differences associated with program activities and procedures, both types of treatments were designed to be completed by the patients at home, with the aim to limit family burden associated with visiting a clinic and to ensure easier access to rehabilitation treatments.

In keeping with aforementioned reviews, we included both randomized and nonrandomized controlled studies: in fact, previous research found that these two study procedures produce similar effect sizes [30, 31]. The search strategy we adopted to detect eligible records led us to include studies that were not considered in any previous reviews, ensuring a more comprehensive overview of remote interventions for pediatric ABI. A further innovative characteristic of this work is that we also performed a meta-analytic exploration on those studies including a control group, in order to provide an overall estimation of the mean effect of TP. Such an investigation allowed us to obtain evidence-based data on treatments for pediatric ABI, which has been reported to be a need by the Recommendations for Research of the Centers for Disease Control and Prevention [32].

## 2. Method

### 2.1. Study Selection for Review and Meta-Analysis

A literature search was conducted using scientific online databases (PubMed and Scopus) to identify pertinent studies, using the terms: *brain injury* AND (*rehabilitation* OR *training*) AND (*home* OR *school*) and (*children* OR *pediatric* OR *adolescents*). The period of time considered for study inclusion ranged from January 2000 to December 2018. We excluded studies published before 2000 to limit the search to more advanced technology-based programs. Additionally, the published article databases of Cogmed Working Memory Training, Lumosity Cognitive Training, and Brain Training software programs were browsed through their websites. Reviews on the topic obtained from search process on PubMed and Scopus were screened to minimize the risk of overlooking or missing out potentially suitable studies for inclusion [12, 14–17].

Criteria for inclusion for study selection were the following: (i) children and adolescents (aged < 21 years old) (US Department of Health and Human Services Food and Drug Administration Guidance for Industry and FDA Staff: Pediatric Expertise for Advisory Panels. Rockville, MD: US Department of Health and Human Services, Food and Drug Administration, Center for Devices and Radiological Health; 2003.); (ii) diagnosis of ABI (e.g., TBI, brain infection, anoxia, and cancer-related injury); (iii) usage of remotely delivered technology-based intervention programs; (iv) limited intervention of clinicians (no more than 1 hour per week); (v) performance-based cognitive measures or rating-scale behavioral indexes as primary outcomes for TP addressing cognitive or behavioral aspects, respectively; and (vi) articles published in peer-reviewed English-language journals.

Only studies with a group-controlled design and reporting immediate post-training outcomes were considered for inclusion in the meta-analytic exploration (the study by Treble-Barna and colleagues [33] compared an experimental group with sex- and age-matched healthy controls performing the same training. Thus, given the different diagnoses and the lack of a passive control group or an active control group performing a low-demanding training, this study was not included in the meta-analytic examinations). If an article reported both immediate post-treatment and follow-up measures, only immediate post-training outcomes were selected. Congruently, if an article reported follow-up data of a previous study, only data from the first published study was extracted. This choice was made as some studies either did not report any follow-up measures at all or reported follow-up measures taken at different time points since treatment completion. This limits heterogeneity, as effects associated with receiving a treatment may change over time.

We excluded from both the review and the meta-analytic exploration studies: (i) applying rehabilitation programs with an exclusive focus on motor skills, (ii) reporting feasibility and acceptability measures only, (iii) assessing the effects of interventions tailored to parents, (iv) assessing the effects of interventions tailored to children and parents, but reporting outcomes of only parents; (v) clinician-delivered, and (vi) testing the usage of external aids (reminder messages, diaries, etc.). On a related note, the study of de Kloet and colleagues [34] included a subgroup of participants exceeding the age limit. However, since the majority of patients ranged between 8 and 18 years old, we decided to include the study in this work. For those studies on behavioral TP reporting outcomes on both children and parents, we collected only outcomes related to children.

The extent of support provided by clinicians during the rehabilitation practice was case-wise evaluated to check study eligibility. If an intervention implied weekly contact with clinicians to assess adherence and/or resolve any type of problems that occurred in performing the TP, it was evaluated as eligible. Otherwise, if a training required the presence and continuative support of a clinician, it was not considered for inclusion. Three studies [34–36] indicated clinic as one of the possible settings where the intervention could be held, but they described interventions primarily feasible for ecological settings (i.e., home or school) and were, thus, evaluated eligible for inclusion in this work.

For the meta-analysis on cognitive TP, we considered only performance-based measures related to cognitive domains, thus excluding outcomes associated with behavioral, social, and emotional functioning, quality of life, and everyday cognitive functioning. This is because we were interested in examining only the efficacy of the programs on cognitive domains as assessed through performance-based measures, which constituted the specific targets of the interventions.

**Figure 1:**
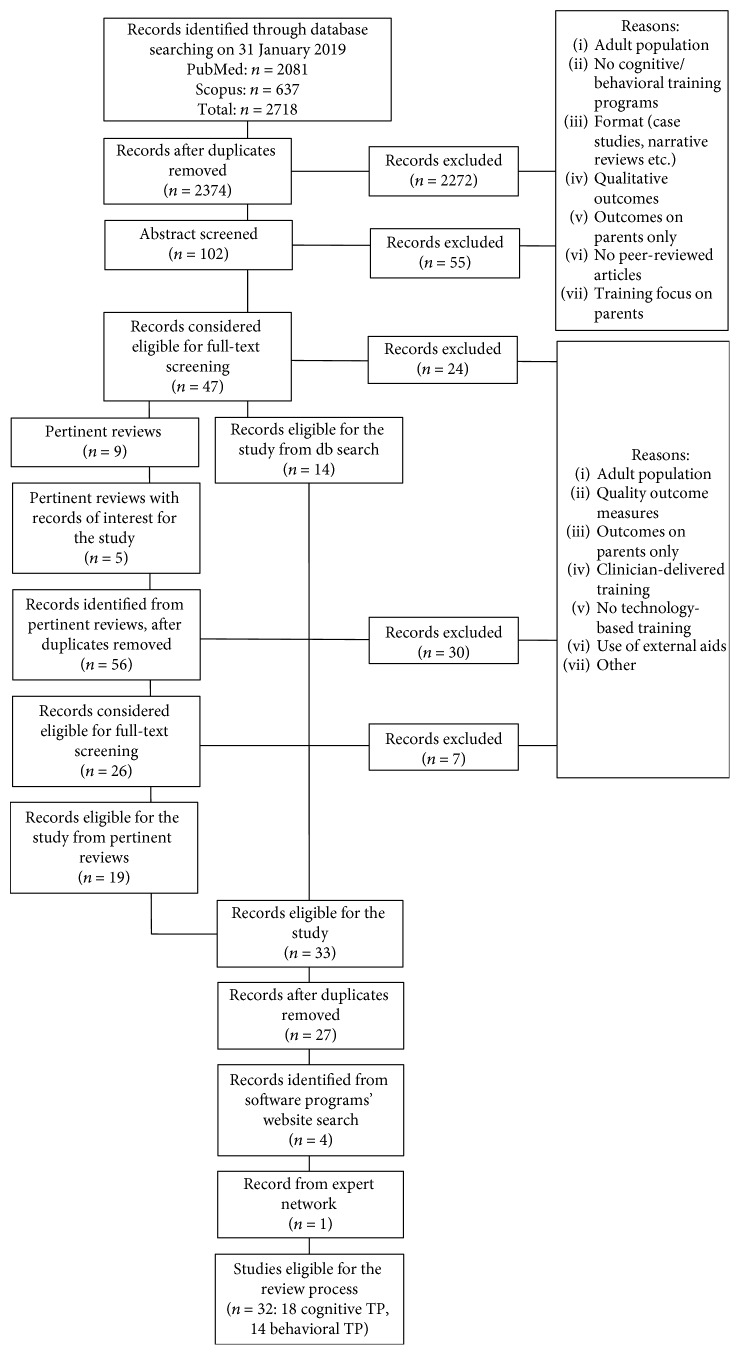
Graphical depiction of the study inclusion procedure.

The literature search identified a total of 2,718 records on 31 January 2019. 637 records were collected from Scopus and 2,081 from PubMed databases. After the removal of duplicates (*n* = 344), titles of 2,374 records were screened. Based on the title, 2,272 records were excluded due to irrelevant content (e.g., adult populations and no cognitive/behavioral training) or format (e.g., case reports and narrative reviews). Of the remaining 102 records, the abstract screening led to 47 records considered to be eligible for full-text screening. Among the selected 47 records, 24 records were excluded due to irrelevant content or format, 9 records were reviews, and the remaining 14 records were studies eligible for this work. From the review screening, 4 reviews were considered to be not relevant, as they did not include technology-based training [19, 20] or were published in 2009-2010 [1, 18]. Thus, most recent reviews were preferred for study search [12, 14–17], after controlling that they included studies also reported in less recent reviews. 75 records were identified from the 5 pertinent reviews, of which 19 were duplicates. Of the 56 remaining records, 26 were considered to be eligible for full-text screening. Of these 26 records, 2 were excluded as the training was not home-based or required direct tutoring [37, 38] and 4 other records were removed as they applied the Amsterdam Memory and Attention Training for Children (AMAT-c [39–42]), which mostly proposes paper and pencil exercises [43]. Therefore, a total number of 19 records were obtained from the review screening. However, 6 records identified from review screening were duplicates of records identified from database searching. Therefore, after removing duplicates, a final number of 27 eligible studies was obtained from database search and review screening. Moreover, 4 records from software programs' website [44–47] were found. One more article meeting the eligibility criteria was found from expert network [48], leading to a total of 32 studies considered for this work.  Figure 1 depicts the study inclusion procedure.

### 2.2. Meta-Analytic Exploration: Data Extraction and Analysis

For studies inserted in the meta-analytic exploration, sample size, mean, and standard deviation (SD) of the primary outcomes were extracted for both the intervention and the control group. If only error of the mean (SE) was provided, SD was calculated by applying the following formula SD = SE×√*n*. For data presented graphically, we used the free online WebPlotDigitizer software to extract them from graphs (http://arohatgi.info/WebPlotDigitize). Moreover, first authors were contacted by e-mail if relevant data were not reported in the article.

Since all the studies delivering either cognitive or behavioral TP provided multiple outcome measures, we chose to adopt two distinct approaches to compute the observations to be inserted in the meta-analytic examination (see [49]).

With respect to cognitive TP, outcome measures of cognitive abilities extracted from the articles were classified into four main domains: (i) visuo-spatial skills, including visuo-spatial working memory and visuo-spatial attentional tasks; (ii) executive functions, including inhibition, planning, and cognitive flexibility tasks; (iii) math skills, including arithmetic operations and problems; and (iv) verbal skills, including verbal working memory, semantic/phonological processing, and reading tasks. A first meta-analytic model (model Cognitive A) included, for each study, an aggregated mean representing the mean value of the various outcomes related to all cognitive domains (namely, executive functioning, visual-spatial working memory, language, and math). In contrast, a second model (model Cognitive B) included, for each study, the mean of the outcomes of the cognitive domain that yielded the highest effect size. Indeed, since the primary outcome domain of studies was not always clearly identifiable, this alternative approach seems suitable if we assume that the outcome domain with the highest effect size may represent the cognitive function gaining the most beneficial effects from the TP, regardless of the authors' expectations (whether stated or not in the articles).

With respect to behavioral TP, a first model (model Behavioral A) included, for each study, an aggregated mean representing the mean value of the outcomes of the two assessed behavioral domains (namely, everyday executive functions and behavioral, social, and emotional functioning) combined together or the mean score of the only assessed behavioral domain. The mean score of the “behavioral, social, and emotional functioning” domain was calculated as the mean of the indexes/subscales of Child Behavior Checklist (CBCL), Home and Community Behavior Scale (HCSBS), and Behavioral and Emotional Rating Scale 2 (BERS 2). In case both parent- and child-compiled questionnaires were reported, we calculated an aggregated mean of the two scores for each index or subscale. The mean score of the “everyday executive function” domain was obtained by the score of the Global Executive Composite (GEC) scale of the Behavior Rating Inventory of Executive Function (BRIEF) questionnaire, which was the only instrument adopted to evaluate this area throughout the included studies. In contrast, a second meta-analytic model (model Behavioral B) included, for each study, the mean of the behavioral domain that yielded the highest effect size, which was considered to be the area gaining the most beneficial effects from the TP.

Whenever a study reported results divided per subsamples of participants according to different characteristics (e.g., age range and severity of the diagnosis), the weighted mean [(*μ*_1_∗*n*_1_ + *μ*_2_∗*n*_2_+⋯)/*k*] and pooled SD [√(SD_1_^2^ + SD_2_^2^+⋯+SD_*n*_^2^)/*k*], where *k* = total number of participants per subsample, were calculated for each measure. Moreover, if a study reported both cognitive and behavioral measures, the outcome to be inserted in the model was chosen according to the focus of the intervention program. Therefore, an aggregate mean of all cognitive measures was calculated for studies applying a training with a focus on cognitive skills [35, 36, 46, 47, 50–52], whereas an aggregate mean of questionnaires and rating-scale scores was chosen for studies focusing on behavioral competences [23, 53–58]. For cognitive TP, performance-based measures were preferred over self-report scales, since only 4 out of the 7 included studies administered questionnaires. Moreover, adding questionnaire scores to performance-based measures would have introduced potential sources of heterogeneity. In a study on behavioral TP [57], three groups of participants were compared: a group performing traditional Teen Online Problem-Solving (TOPS-F), a group performing a modified version of the TOPS (TOPS-TO), and a control group assigned to Internet Resource Comparison (IRC). As the Authors reported that study participants were recruited as part of a randomized clinical trial between March 25, 2010, and August 6, 2014, and data on TOPS-F were reported in some previous studies [22, 23, 58], we decided to consider for the meta-analytic investigation only the comparison between TOPS-TO and IRC. Hedges's *g* was calculated for each study using the following formula:

(1) $$\begin{array}{c} g=\frac{\left(M1-M2\right)}{\left(\text{SD}*\text{pooled}\right)}. \end{array}$$

Hedges's *g* is often referred to as a corrected effect size as compared to Cohen's *d*, as it corrects for a bias in the effect size due to the inclusion of studies with small samples [59]. The obtained effect sizes were then tested in random effects models. These models were preferred over a fixed-effect model due to the heterogeneous methodology applied across studies. Assessment of heterogeneity of the effect sizes was evaluated by calculating *Q* statistic, which indicates if the variance of the estimate effect sizes is greater than to be expected from sampling error, suggesting that the observed variance may be explained by other factors beside the intervention. Additionally, *I*^2^ ratio, describing the percentage of variation across studies that is due to true heterogeneity rather than chance, was calculated to quantify and evaluate the consistency of the findings [60].

Finally, a nonparametric rank-order correlation between estimate effect sizes and sample sizes of the studies was calculated for the models Cognitive A and Behavioral A examining aggregate means of all outcome domains [61]. Indeed, a negative correlation between the two measures can be interpreted as a proxy for the presence of publication bias (for details see [62]).

A significance level of *p* < .05 was used for all analyses. Analyses were performed with Meta and Metafor software packages [63].

## 3. Results

### 3.1. Results from the Review Process

#### 3.1.1. Cognitive Training Programs

18 studies (see Table 1) investigated 7 different remote cognitive TP consisting of drill-based exercises aimed at stimulating different cognitive functions: TherapWii Protocol [34], Cogmed Working Memory Training [44–47, 50, 64], Lumosity Cognitive Training [21, 29], Captain's Log [35, 36, 65], Attention Improvement and Management (AIM) Program ([33, 66]), Brain Games Program [67, 68], and “Move it to improve it” (Mitii^TM^) Protocol [51, 52]. Alongside exercises targeting cognitive functions, the AIM Program additionally proposed metacognitive strategy use, while the Mitii Protocol proposed web-based multimodal exercises, based on perceptual and motor abilities. The cognitive domains trained by the different programs were: attention, working memory, short- or long-term memory, language comprehension, executive functioning, processing speed, and math. All programs addressed specific cognitive functions, except the TherapWii Protocol, which was aimed at ameliorating the global physical, mental, and social functioning of the children and for which the intervention objectives in the various life domains were set on the basis of the areas in which the children exhibited an impairment. However, the majority of patients (47/50) recruited for receiving TherapWii Protocol [34] presented with problems on information processing and all of them received cognitive exercises. Moreover, in 3 studies [34–36], the TP could be performed not only in the ecological setting (home or school) but also in an individual rehabilitation setting with a therapist [34] or in a clinic without the presence of a therapist [35, 36]. The other programs were solely home-based.

Before training onset, patients usually received detailed instructions on how to perform exercises by a clinical researcher, usually in the hospital setting. In 2 studies [33, 66], the training protocol required patients to reach the clinical center to meet the therapist once a week to monitor performance and progress and to discuss the cognitive strategies to adopt in the tasks administered the following week. In 2 studies [35, 36], no therapist monitoring was provided, while in 2 studies [67, 68] participants received a calendar and a training schedule to self-monitor their own adherence and performance. In the remaining 12 studies, remote weekly contact (by phone or email) was provided by a therapist to monitor adherence and resolve possible problems encountered by participants.

The TP had a variable duration of 5-20 weeks, with a commitment varying from 2 to 6 days per week. In 2 studies [34, 65], the specific number of sessions per week was not defined, but a minimum weekly time engagement was required, which ranged from 50 minutes to 2 hours per week. All TP required only the use of a computer with Internet access, except the TherapWii Protocol, which required also the use of a Nintendo Wii, the Mitii Protocol, which required also the use of a Kinect to track gross motor or upper limb movements, and a version of Brain Games platform which was delivered through iPad. The difficulty of the tasks provided in the interventions was calibrated on patients' abilities in 13 out of 18 studies. Specifically, Brain Games, Cogmed Working Memory Training, Lumosity Cognitive Training, and Captain's Log were adaptive programs, automatically modifying the difficulty of the exercises based on the individual performance. Similarly, most cognitive exercises of the TherapWii Protocol [34] were adaptive. In contrast, the menu of drills and strategies of the AIM Program was initially defined by a clinician on the basis of neuropsychological testing, parent and child reports, and progressively updated by a therapist during training in accordance with changes in performance [33, 66]. With respect to the Mitii Protocol, the complexity of exercises was adjusted weekly by a trainer based on performance and comments provided by the child and family [51, 52].

With respect to study methodology, the sample size of studies varied between *n* = 5 and *n* = 68, with 5 studies including < 20 total participants [33, 64–66, 68]. Nine studies [29, 35, 36, 45–47, 50–52] had an active or a passive control group of children with ABI performing low-demanding TP or no training, respectively. In contrast, a study [33] had a control group of healthy children performing the same experimental training. Participants had ABI of both traumatic (TBI) and nontraumatic nature (e.g., stroke, brain tumor, and cancer-related treatments). One study [29] included also patients with congenital brain damage (*n* = 4).

Regarding inclusion criteria, studies were generally conducted on patients without sensory or motor deficits that could interfere with participation in the TP. Moreover, some studies [44–47, 50, 64] established a criterion based on IQ, excluding patients with clinically impaired or borderline intellectual functioning. Some other studies [34, 51, 52] excluded individuals with severe cognitive impairment, but without declaring a specific IQ threshold. In contrast, another study [29] included patients with very low IQ to test the feasibility of the selected TP among the general population of children with brain damage, who displays heterogeneous cognitive functioning. Moreover, 5 studies [21, 33, 44, 47, 65, 66] reported as inclusion criterion the presence of specific cognitive deficits in attention and/or memory abilities or executive functions, as measured through neuropsychological tests and/or parents' reports.

The measures used to test cognitive outcomes were all standardized in 16 out 18 studies. In contrast, in the study of Corti et al. [29], which was aimed at assessing feasibility as primary objective, only preliminary results on training effectiveness were indicated. These results were based on a nonstandardized index directly supplied by the cognitive TP and indicating the average change in performance between the first and the last training day, which could be considered as a near-transfer measure. Linden and colleagues [67] used both standardized tests and nonstandardized computerized tasks. Two studies out of 18 [51, 52] reported no improvement. In the other 16 studies, improvements after training were found both in near-transfer and/or far-transfer measures (e.g., academic achievement and everyday functioning), at least at post-training assessment.

#### 3.1.2. Behavioral Training Programs

With respect to technology-based TP aimed at improving behavioral issues, a series of 14 studies conducted between 2005 and 2018 by the same research group in the USA were included in this work (see Table 2). 12 studies investigated the efficacy of family web-based interventions aimed at improving executive, behavioral, and social functioning of children with TBI. In particular, 6 studies focused on the Counselor-Assisted Problem Solving (CAPS) intervention [53, 54, 56, 69–71], 2 studies on the Online Family Problem-Solving (OFPS) program [55, 72], and 4 studies on the Teen Online Problem-Solving (TOPS) intervention [22, 23, 57, 58]. The remaining 2 studies focused on the Social Participation and Navigation (SPAN) intervention [48, 73], a training aimed at improving social abilities in children with TBI or brain tumor.

The CAPS, OFS, and TOPS programs consisted of self-guided, online sessions performed by the child and his/her family at home and of Skype videoconferences with a coach with expertise in cognitive-behavioral therapy. Such videoconferences were included with the purpose of reviewing the exercises completed by participants and providing feedback on the implementation of the problem-solving process in real life. Specifically, the training was composed of 8-10 core sessions focusing on executive functions, social skills, injury-related issues, and other eventual supplemental sessions. Participants were advised to perform supplemental sessions only if their specific content was evaluated potentially beneficial for the ongoing problems of the child and/or the family at the baseline assessment.

The SPAN program, together with online didactic sessions, included a dedicated iPhone application to set children social goals and weekly videoconferencing sessions (via Skype) with a trained college-student coach. The introduction of a peer as a coach instead of the therapist was motivated by the fact that the impact of peers becomes increasingly relevant through adolescence [48, 73].

In some studies [22, 23, 55, 58, 72], meetings with the coach [48, 73] were scheduled every 1-2 weeks, whereas in other studies [53, 54, 56, 57, 69–71] they were scheduled every 2-4 weeks. The CAPS, OFS, and TOPS program duration could approximatively range from 6 months to 8 months, based on the total number of sessions assigned to participants. The SPAN program duration ranged from 4 weeks [73] to 10 weeks [48].

With respect to study methodology, the sample size of studies varied between *n* = 4 and *n* = 132, with 4 studies including < 20 families [22, 48, 72, 73]. In the early studies, participants were aged between 5 and 16 years [55, 72], while in the subsequent studies they were only adolescents [22, 23, 48, 53, 54, 56–58, 69–71, 73]. Only one study extended to 22 years the age range of participants [48]. In all studies, children were included if they sustained a moderate-to-severe TBI about 1-24 months before the intervention. Only a study [48] included also participants with a diagnosis of brain tumor.

In the majority of the studies, children who experienced blunt trauma or history of abuse and had familiarity for psychiatric hospitalization were excluded. In those studies that were conducted between 2013 and 2016 [53, 54, 56, 69, 71], the presence of intellectual disability before the injury was set as a further exclusion criterion.

Eleven out of the 14 studies included in this work were randomized controlled trials. In 10 studies [23, 25, 53–56, 58, 69–71], the children and their families could be randomly assigned to one of the TP described above (CAPS, OFPS, or TOPS) or to an Internet Resource Comparison (IRC), used as a control condition activity. In one study [22], the control group received the TOPS intervention void of audio content.

The efficacy of the interventions was assessed through parent-report or self-report questionnaires evaluating everyday executive functions and behavioral, social, and emotional functioning. With respect to outcomes on children, all studies reported positive gains from the interventions in the various domains assessed. Results also revealed that these treatments reduced parent-teen conflicts.

### 3.2. Results from the Meta-Analytic Exploration

**Figure 2:**
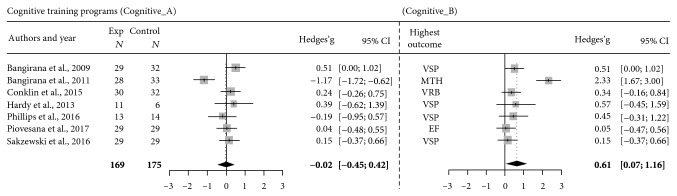
Forest plots showing the number of participants per group, effect size estimates (Hedges's *g*), and 95% confidence interval of each study, comparing cognitive training outcomes with control condition's outcomes. Model Cognitive A depicts the meta-analytic model that considers, for each study, a combined mean value representing the mean value of multiple outcomes relative to all cognitive domains. Model Cognitive B depicts the meta-analytic model that considers, for each study, the highest outcome domain. Note: VSP = visuo-spatial skills; MTH = math; VRB = verbal; EF = executive functioning.

The first random effects model on cognitive TP (Cognitive A), including the aggregate means of all reported outcomes of the 7 eligible studies, yielded a nonsignificant estimated effect size of -.02, 95%CI = −0.45; 0.42, *Z* = −0.08, *p* = .94 (Figure 2). Total heterogeneity of the effect sizes was significant as indicated by *Q* = 23.32, *p* < .001, and *I*^2^ = 74.3% (45.1%-87.9%), revealing the presence of high methodological and/or statistical heterogeneity between studies.

The model Cognitive B, including the measures returning the highest effect sizes in cognitive training studies, yielded a significant estimated effect size of .61, 95%CI = 0.07; 1.16, *Z* = 2.19, *p* = .03 (Figure 2). Total heterogeneity of the effect sizes was significant, as indicated by *Q* = 34.7, *p* < .0001, and *I*^2^ = 82.7% (65.7%-91.3%), confirming the presence of high methodological and/or statistical heterogeneity between studies.

**Figure 3:**
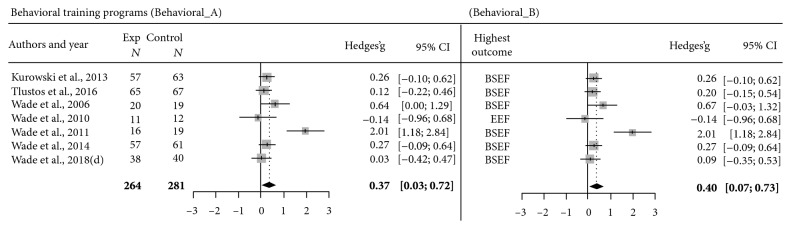
Forest plots showing the number of participants per group, effect size estimates (Hedges's *g*), and 95% confidence interval of each study, comparing behavioral training outcomes with control condition's outcomes. Model Behavioral A depicts the meta-analytic model that considers, for each study, an aggregated mean representing the mean value of the various outcomes relative to all behavioral domains. Model Behavioral B depicts the meta-analytic model that considers, for each study, the highest outcome domain. Note: BSEF = behavioral, social, and emotional functioning; EEF = everyday executive functioning.

Regarding the behavioral TP, model Behavioral A, including the aggregate means of all reported outcomes, yielded a significant effect size of .38, 95%CI = 0.03‐0.72, *Z* = 2.15, *p* = .03 (Figure 3). High heterogeneity was detected by *Q* = 20.95, *p* < .002, and *I*^2^ = 71.4% (37.8%-86.8%).

The last model, Behavioral B (Figure 3), including the highest outcome of each of the 7 behavioral training studies, yielded a significant effect size of .39, 95%CI = 0.07‐0.73, *Z* = 2.35, *p* = .02. High heterogeneity was detected by *Q* = 19.87, *p* < .003, and *I*^2^ = 69.8% (33.7%-86.2%).

The subgroup analysis conducted on the model Cognitive B on the outcomes associated with the greatest improvement showed a significant effect size of .37, 95%CI = 0.06‐0.69, *Z* = 2.34, *p* = .02, for the subgroup of measures assessing visuo-spatial skills (*k* = 4) (the remaining three outcomes referred each one to a different cognitive function—i.e., executive function, math, and verbal skills), accompanied by a sensible reduction in total heterogeneity as detected by both *Q* = 1.19, *p* = .76, and *I*^2^ = 0.0% (0.0%; 61.5%).

Lastly, the nonparametric rank-order correlation between the effect size estimates and sample size across studies was nonsignificant for both model Cognitive A (*r* = −.09, *p* = .84) and model Behavioral A (*r* = −.39, *p* = .39), showing no clear evidence for publication bias.

### 3.3. Risk of Bias

In order to assess the validity and evaluate potential methodological problems of the included studies, two researchers (VO and EF) screened the articles on cognitive TP and two researchers (CC and CO) screened the articles on behavioral TP. The evaluation of risk of bias in this work was applied to all included studies, not only to clinical trials, in order to provide a detailed overview of methodological study characteristics. For this purpose, the researchers considered the domains included in Risk of Bias Tool of The Cochrane Collaboration [60]. This tool provides a guide to assess methodological quality of clinical and research trials according to the following five domains: (i) selection bias, which refers to the method of allocation to treatment condition, considering both sequence of randomization (unpredictable or not) and adequate concealment of the allocation sequence from those involved in the enrollment and assignment of participants; (ii) performance bias (potential cause of expectation and placebo-induced effects), deriving from unsuccessful blinding of participants and personnel to treatment allocation; (iii) detection bias, deriving from unsuccessful blinding of outcome assessors to participants' treatment allocation that may influence data collection and analysis; (iv) attrition bias, usually detected in the presence of a not justified high proportion of missing outcomes due to participant drop-out (attrition) during the study or exclusions from the analysis, causing a possible observed biased effect estimate. Attrition could be due to participants' withdrawal, missing of a scheduled appointment at which outcomes should have been measured, unsuccessful data collection during an appointment, failing to complete questionnaires, loss to follow-up associated with the fact that participants cannot be located, follow-up ceasing by researchers, and loss of data and records; and (v) reporting bias, indicating the risk that non-significant results may be withheld from publication due to selective omission of outcomes from reports, selective choice of data for an outcome, selective reporting of analyses using the same data, selective reporting of subsets of the data, and selective underreporting of data. The absence of a registered protocol or the presence of a registered protocol without an exact description of instruments used to assess outcomes could represent a potential risk of reporting bias. The risk associated with each assessed domain was judged as low, high, or unclear. In case that such an evaluation was not applicable, this was indicated. The researchers provided judgements on risks of bias for all studies independently and then reached a final agreement in case of discrepancies. Results are summarized in Table 3.

With respect to cognitive TP, selection bias associated with random sequence was judged low for 52.9% of studies, while the other half had an experimental design that could not allow the assessment of sampling method. Selection bias associated with allocation to treatment methods was judged low for 16.6% of the studies, high for 11.1%, and unclear for 22.2%. For the remaining studies (50.1%), this criterion was not assessable. Performance bias associated with blinding of participants and personnel procedure was associated with low risk of bias for 22.2% of the included records, whereas 16.6% was judged at high risk of bias and 11.1% was defined as at unclear risk of bias. For the remaining 50.1% of studies, this criterion was not assessable. Detection bias associated with blinding of assessor procedure was associated with low risk of bias in 44.6% of the studies, high risk of bias in 27.7%, and unclear risk of bias in 27.7%. Attrition bias associated with incomplete outcome data was associated with low risk of bias in 66.7% of the studies, with the remaining 33.3% being associated with high risk. Reporting bias associated with selective reporting was associated with unclear risk of bias in 100.0% of the studies.

Considering behavioral TP, selection bias associated with random sequence was judged low for 71.4% and unclear for 7.1% of the studies, while for the remaining 21.4% it was not assessable. Selection bias associated with allocation to treatment methods was judged low for 35.7% of the studies, high for 23.5%, and unclear for 14.3%. For the remaining 21.4%, this criterion was not applicable. Performance bias associated with blinding of participants and personnel procedure was associated with high risk of bias for 78.6% of the included records, whereas for the remaining 21.4% this criterion was not applicable. Detection bias associated with blinding of assessor procedure was associated with low risk of bias in 42.9% of the studies, high in 28.6%, and unclear in 28.6%. Attrition bias associated with incomplete outcome data was associated with low risk of bias for 21.4% of the studies, high risk for 21.4%, and unclear for 57.2%. Reporting bias associated with selective reporting was associated with high risk of bias for 14.3% of the studies and unclear for the remaining 85.7%.

## 4. Discussion

This review described 32 technology-based TP (training programs) for pediatric patients with different types of ABI delivered in the ecological setting, of which 18 were cognitive TP and 14 behavioral TP. A meta-analytic exploration was also conducted on a subsample of 14 studies applying a control-group design (7 cognitive TP and 7 behavioral TP), in order to provide an estimation of the overall mean effect of the eligible studies. The presence of a control group of participants should be considered as an essential methodological element in studies investigating treatment efficacy [32], as it allows controlling for the effects of spontaneous development in pediatric age and possible expectation bias. The picture offered by this review indicates that, nowadays, about half of the studies on remote TP addressing cognitive functions for pediatric ABI lacks a control on these aspects. With respect to behavioral TP, most of the studies included a control group, thus being in line with the methodological recommendations of evidence-based research.

Based on the review process, 16 out of the 18 studies on cognitive TP were found to be effective in improving cognitive outcomes immediately after training, on both near- and far-transfer measures. Moreover, 8 out of the 12 studies on cognitive TP that considered also outcomes on functioning in daily life found positive gains. This would suggest that computer-based interventions offering drill-based exercises delivered in an ecological setting are effective at improving cognitive abilities and adjustment in children with ABI. Nevertheless, data of our meta-analytic exploration seem to suggest that cognitive TP are unlikely to have a positive impact on different cognitive domains, suggesting limited generalized effects. Indeed, the meta-analysis conducted on the combined mean of all cognitive outcomes of studies showed no effect (model Cognitive 1). In contrast, the meta-analytic model on the cognitive domain associated with the highest improvement for each study (model Cognitive 2) yielded an effect of moderate level (*g* = 0.61), according to criteria taken from previous literature [74]. This points to the efficacy of cognitive TP in producing benefits limited to specific cognitive domains, probably those that are mostly stimulated by training tasks. Nevertheless, a high level of heterogeneity (*I*^2^ = 83.2%) was detected. Such a high heterogeneity could be related to various factors associated with characteristics of the samples (e.g., age range, diagnosis subcategory, and time from injury) or of the TP (e.g., focus, duration, adaptation of exercise difficulty, and trained cognitive domains). An additional explorative analysis, performed to evaluate potential differences in TP effects across cognitive domains, highlighted that measures assessing visuo-spatial skills, which represented the highest outcome domain in 4 out of 7 studies, yielded a small, yet significant estimate effect size. This result was accompanied by a low level of heterogeneity, thus indicating that it could constitute a more precise estimation of cognitive TP effects. The improvement in visuo-spatial skills may be due to an intensive stimulation of visuo-spatial processes throughout all TP tasks, which is in accordance with data from previous research indicating that video- and computer-game playing stimulate visuo-spatial abilities, even after a limited training duration [75–77]. For outcomes related to executive functions, verbal and math skills, we could not evaluate the overall training effects, as only one study per domain was available.

On a related note, it is important to stress that the limited amount of studies on cognitive TP eligible for inclusion in the meta-analysis did not allow us to examine in more details the impact of factors that may explain the variability emerged across studies (e.g., training adaptation, duration, match between training focus and primary study outcome). Future research should explore these aspects to identify and weigh their contribution to the success of interventions.

With respect to behavioral interventions, 14 studies from the same team of researchers in the USA were included in this work. Based on the review process, results on these programs are encouraging, as all studies found improvements in everyday executive functioning and/or externalizing behaviors. In some studies, gains related to internalizing symptoms were found too. The meta-analytic model conducted on the combined mean of all behavioral outcomes of studies (model Behavioral A) yielded an estimate effect of small-to-moderate level (*g* = 0.37), which points to positive effects of behavioral TP. This effect was confirmed also by the meta-analytic model considering the outcome associated with the highest improvement (*g* = 0.40; model Behavioral B). A possible explanation for this finding is that the different questionnaires assessing behavioral competences comprised some items evaluating similar behaviors, irrespective of the fact that they were aimed at assessing everyday cognitive functioning, psychological adjustment, or social behaviors. Thus, the selection of the behavioral domain generating the highest effect did not have an impact on results, in contrast to what was observed for cognitive TP.

As aggressive behavior, mood lability, and social isolation have been frequently reported for patients with ABI and appear to increase with age [11, 78], the remote behavioral TP reviewed in the present work may constitute valid alternatives to rehabilitation treatments on these aspects. The success of behavioral TP could be in part attributed to the direct and strong involvement of family members, which recurred in 6 out of the 7 studies included in the meta-analytic exploration. Indeed, family involvement seems to promote generalization of the effects of rehabilitation interventions on children [55, 79, 80]. Nevertheless, one study focused on a behavioral training providing support of peers instead of involvement of family members [57]. Thus, a continuative and structured involvement of other agents, not specifically confined to relatives, may be associated with successful effects of behavioral telerehabilitation.

Nevertheless, also for behavioral studies, a high level of methodological heterogeneity emerged from statistical analyses, despite TP methodology and sample diagnosis were similar across the various studies. Thus, total heterogeneity could likely be due to differences in specific demographic or clinical characteristics of enrolled children or families. This explanation seems to be in line with indications provided by the members of the research team studying behavioral TP [24–26], suggesting that it could be worth for future research to weigh the impact of those specific variables related to the children or the families that may moderate training efficacy. Such an investigation could allow identifying the specific characteristics of individuals that are more likely to benefit from behavioral TP.

It is worth noting that the efficacy of behavioral TP was assessed through questionnaires, which may be affected by expectation bias associated with the fact that patients/parents know they/their children are receiving a treatment and thus may overestimate changes after the intervention (e.g., [81, 82]). This expectation bias can be controlled with a successful blinding. However, given the nature of the included studies on behavioral TP, participants could not be blinded from group assignment (see [15]). In this regard, the introduction of performance-based measures may represent a suitable option to consider for future research with the aim to assess the behavioral effects of these TP and, at the same time, to ensure better methodological procedures and enhance validity. Nowadays, performance-based assessment to investigate everyday executive function behaviors [83, 84] or sociocognitive functioning through virtual reality tools [85, 86] has been described in the relevant literature, thus representing a feasible method to adopt.

### 4.1. Limitations of This Review

In this work, we included research on TP only published in English, which could have led to the omission of relevant research on this issue. Moreover, even though our search strategy was thought to minimize the risk to omit relevant research, as it was based on (i) adoption of two widely used comprehensive databases, namely PubMed and Scopus, (ii) identification of studies reported in recently published meta-analyses and reviews on the topic, and lastly, (iii) search of relevant research on the websites of the main TP, it is still possible that some pertinent records may have been missed out. Second, study heterogeneity was high for both cognitive and behavioral TP, which could have affected results and limited the generalizability of conclusions. The relatively small number of studies inserted in the meta-analytic examinations prevented the possibility to conduct more detailed subgroup analyses aimed at exploring the contribution of specific variables on outcomes, leaving to future research the interesting task of clarifying which components of remote TP lead to the best effects. On a related note, data of this meta-analysis are limited to those studies having a control group, thus representing only a half of the studies on TP addressing cognitive functions. Finally, the evaluation of training efficacy in this work was confined to post-training outcomes, thus preventing any conclusions on long-term effects.

## 5. Conclusions

In summary, results of the present work suggest that remote computer-based TP with a cognitive or a behavioral focus for pediatric ABI may be effective in improving children's functioning. This was confirmed both by the review process and by the meta-analytic investigation. The possibility to address cognitive and behavioral impairments by delivering home-based treatments should be considered as a relevant opportunity for the rehabilitation course of these children. Indeed, TP delivered in ecological settings may help overcome the limited availability of services outside the clinical centers or the urban areas and limit the time and economic demands to families associated with reaching the hospitals [12, 29]. Nevertheless, the high methodological heterogeneity of studies prevents us from drawing definite conclusions. Future research is required to verify the effects of remote TP addressing cognitive and behavioral functions in specific populations of children with ABI based on precise etiology, age range, intellectual competence, and family socioeconomic status. However, despite methodological limitations, a consistent finding of this study is the improvement of visuo-spatial abilities after a computerized intervention addressing cognitive functions. This result is in accordance with data reported in the literature on healthy individuals [75–77] and suggests that the usefulness of drill-based computerized cognitive interventions to enhance visuo-spatial abilities also occurs in case of damage to a developing central nervous system.

## Figures and Tables

**Table 1:** Overview of the studies on Cognitive Training Programs.

Study	Research design	Sample	Treatment characteristics	Treatment domain(s)	Outcome measures	Main findings
Bangirana et al., [36]^∗∗^	Pre/posttest design. Control group: passive control group.	*N* = 65 (E: 32, C: 33). Age: E: mean age = 10.09 years (SD = 2.64 years); C: mean age = 9.71 years (SD = 2.28 years). Diagnosis: cerebral malaria, at approximatively 45 months since injury.	Training: Captain's Log. Description: Captain's Log is a cognitive training aimed at stimulating attention, memory, visuomotor skills, and reason abilities. Fifteen of the 35 possible brain-training exercises of the program were chosen for this study. Training adaptation: the difficulty of exercises automatically increased based on the child's performance. Setting: clinic, home, or school. Duration: 16 sessions in 8 weeks. Frequency: twice a week. Therapist monitoring: not required.	(i) Working memory (ii) Psychomotor speed (iii) Visual attention (iv) Visual learning and memory (v) Spatial working memory and learning (vi) Visuomotor processing speed	*Cognitive functioning*: (i) “CogState” battery: working memory, detection, identification, card learning, maze learning, and maze chasing *Behavioral, social, and emotional functioning*: (i) CBCL (internalizing, externalizing, total problems)	Improvement in (i) Visuomotor processing speed (ii) Working memory (iii) Learning (iv) Internalizing problems (CBCL)

Bangirana et al., [35]^∗∗^	Pre/posttest design. Control group: passive control group.	*N* = 61 (E: 28, C: 33). Age: 5-12 years. Diagnosis: severe malaria at 3 months since injury.	Training: Captain's Log. Description: Captain's Log is a cognitive training aimed at stimulating attention, memory, visuomotor skills, and reason abilities. Fifteen of the 35 possible brain-training exercises of the program were chosen for this study. Training adaptation: the difficulty of exercises automatically increased based on the child's performance. Setting: clinic, home, or school. Duration: 16 sessions in 8 weeks. Frequency: twice a week. Therapist monitoring: not required.	(i) Working memory (ii) Psychomotor speed (iii) Visual attention (iv) Visual learning and memory (v) Spatial working memory and learning (vi) Visuomotor processing speed	*Cognitive functioning*: (i) KABC-II: working memory, visual spatial ability, reasoning, learning, and planning (ii) TOVA *Academic functioning*: (i) WRAT-3: arithmetic, reading, and spelling *Behavioral, social, and emotional functioning*: (i) CBCL (internalizing, externalizing, total problems)	Improvement in (i) Learning mean score

Hardy et al., [65]	Pre/posttest pilot design. Follow-up: at 3 months. Control group: no control group.	*N* = 9. Age: 10-17 years. Diagnosis: 6 BT, 3 ALL at 1-10 years since surgery and/or adjuvant therapies. Inclusion criteria based on cognitive functioning: (i) Presence of attention problems based on CPRS and WMI of WISC 4	Training: Captain's Log. Description: Captain's Log is a cognitive training aimed at stimulating attention, memory, visuomotor skills, and reason abilities. Training adaptation: the difficulty of exercises automatically increased based on the child's performance. Setting: home. Duration: 12 weeks. Frequency: at least 50 minutes of training per week. Therapist monitoring: weekly contact provided by a researcher for motivation and troubleshooting.	(i) Attention (ii) Concentration (iii) Memory (iv) Listening skills (v) Processing speed (vi) Self-control (vii) Hand-eyecoordination (viii) Fundamental numerical concepts (ix) Basic problem solving/reasoning skills	*Cognitive functioning*: (i) WMI of WISC IV (digit-span, letter-numbersequencing) *Behavioral, social, and emotional functioning*: (i) CPRS	Improvement in (i) WMI from baseline to 3-monthfollow-up (ii) Digit-spanforward at post training (iii) Parent-reported attention across the 3 time points

Kesler et al., [21]	One-arm open trial pilot study. Pre/posttest design. Control group: no control group.	*N* = 23. Age: 7-19 years. Diagnosis: 14 ALL, 9 posterior fossa BT, at 6 months since surgery and/or adjuvant therapies. Inclusion criteria based on cognitive functioning: (i) Deficit in EF: two or more EF test scores > 1 SD under the normative mean or the FSIQ score	Training: Lumosity Cognitive Training. Description: Lumosity Cognitive Training is a brain training consisting of different games, each targeting one of the following cognitive domains: speed of processing, attention, memory, flexibility, and problem solving. Only those exercises addressing cognitive flexibility, attention, and working memory (available in 2007 at the beginning of the study) were chosen for the training used in this study. Training adaptation: the difficulty of exercises was automatically and in real time adjusted by the program, based on the child's performance. Setting: home. Duration: 40 sessions in 8 weeks. Frequency: 5 sessions/week, 20 minutes/session, 6 tasks/session. Therapist monitoring: weekly monitoring by research staff.	(i) Cognitive flexibility (ii) Attention (iii) Working memory	*Cognitive functioning*: (i) WISC-IV or WAIS-III (ii) WRAML2 (list memory, picture memory) (iii) NEPSY II (animal sort for children of 7–16 years) (iv) D-KEFS (sorting test for children of 17–19 years) (v) WJ-III(cancellation test) (vi) MVPT-3 *Other measures*: (i) Neuroimaging data	Improvement in (i) Processing speed (ii) Cognitive flexibility (iii) Verbal and visual declarative memory (iv) Prefrontal cortex activation at post training, compared to baseline

de Kloet et al., [34]	Multicenter pre/posttest design. Control group: no control group.	*N* = 50. Age: 6-29 years. Diagnosis: 27 TBI, 23 ABI non-TBI, at 0-2 years since injury.	Training: TherapWii Protocol. Description: this program consists of different Nintendo Wii games, each one composed of several subgames. Training adaptation: the difficulty of the majority of games was automatically adjusted by the program, based on child performance. Setting: home, school, or clinic during an individual rehabilitation session. Duration: 12 weeks. Frequency: at least 20 minutes per week, for a maximum of 2 hours per week. Therapist monitoring: weekly contact provided by a therapist for motivation and troubleshooting.	(i) Working memory (ii) Attention (iii) Information processing (iv) Executive functioning (v) Visuospatial perception	*Cognitive functioning*: (i) ANT *Physical and/or behavioral, social, and emotional functioning*: (i) Time spent on physical activity: ad hoc Likert questionnaire (ii) Social participation: children's assessment of participation and enjoyment *Quality of life*: (i) Peds-QL	Improvement in (i) Alertness (ii) Attentional flexibility (iii) Visuospatial WM (iv) Motor tracking (v) Time on physical activities (vi) Quality of life regarding school and social participation

Hardy et al. [47]^∗∗^	Randomized study. Pre/posttest design. Follow-up: at 3 months Control group: active control group performing the *MegaMemo*, a computer-based program that consists of the same exercises as *Cogmed^RM^*, but the level of difficulty does not increase.	*N* = 20 (9 BT; 11 ALL); (E: 13, C: 7). Age: 8-16 years. Diagnosis: BT and ALL, at 1 year since surgery and/or adjuvant therapies. Inclusion criteria based on cognitive functioning: (i) IQ > 70 (ii) Difficulties with attention and working memory, according to at least one of the following criteria: (1) T-score of inattention subscale of the CPRS-3 ≥ 75th percentile (2) Attention/concentration subscale or working memory indices of the WRAML2 > 1 DS below the mean (3) Attention/concentration subscale or working memory indices of the WRAML2 > 1 DS below the estimated IQ	Training: Cogmed Working Memory Training. Description: this program consists of exercises targeting verbal and visuospatial working memory. Training adaptation: the difficulty of exercises was automatically and in real time adjusted by the program, based on the child's performance. Setting: home. Duration: 25 sessions in 5-8 weeks. Frequency: approximatively 3-5 sessions/week. Monitoring: weekly phone-based contact with a coach.	(i) Visuospatial and verbal working memory	*Cognitive functioning*: (i) WRAML2 (attention/concentration and working memory) *Everyday cognitive functioning*: (i) CPRS (inattention and learning problems scales)	Improvement in (i) Visuospatial working memory (i) Parent-ratedlearning problems

Sohlberg et al., [66]	Pilot study. Pre/posttest design. Control group: no control group.	*N* = 11. Age: 13-16 years. Diagnosis: TBI at >12 months since injury. Inclusion criteria based on cognitive functioning: (i) Attention problems as defined by a frequency score of 2 or 3 on at least 4 out of the 9 items from the Attention Subscale of the Vanderbilt ADHD Diagnostic Parent Rating Scale	Training: Attention Improvement Management (AIM) training. Description: this training is a cognitive intervention aimed at improving attention and executive functions. The program combines computerized drill-based tasks and metacognitive strategy instruction. Training adaptation: difficulty of exercises was weekly adjusted by a therapist. Setting: home. Duration: 10 weeks; the treatment was extended by 1 week for each week that the child failed to complete at least 2 home practices. Frequency: 2-4 sessions/week, 20 minutes/session. Therapist monitoring: home practice sessions were tracked and monitored by a clinician via a USB drive. Weekly face-to-face meetings between the child and the research clinician were scheduled to review home practice and adjust exercise difficulty.	(i) Working memory (ii) Attention (inhibition, sustained, selective/focused attention) (iii) Executive functions (flexibility of thinking, inhibition, problem solving, planning, impulse control, concept formation, abstract thinking, and creativity in both verbal and spatial modalities)	*Cognitive functioning*: (i) TEA-Ch (the Score!, Walk/Do not Walk, Code Transmission, Sky Search) (ii) D-KEFS (Trail Making Test, Color-word Interference Test, and Tower Test) *Everyday cognitive functioning*: (i) BRIEF (GEC) *Other measures*: (i) GAS	Improvement in (i) Attention (ii) Parent reported executive functioning (iii) GAS

Conklin et al., [46]^∗∗^	Single-blind randomized controlled trial. Pre/posttest design. Control group: passive control group.	*N* = 68 (E: 34, C: 34). Age: 8-16 years. Diagnosis: ALL or BT (E: 34, 23 ALL +11 BT; C: 34, 24 ALL +10 BT), at least 1 year since surgery and/or adjuvant therapies. Exclusion criteria based on cognitive functioning: (i) IQ < 70	Training: Cogmed Working Memory Training. Description: this program consists of exercises targeting verbal and visuospatial working memory. Training adaptation: the difficulty of exercises was automatically and in real time adjusted by the program, based on the child's performance. Setting: home. Duration: 25 sessions in 5-9 weeks. Frequency: approximatively 3-5 sessions/week, 30-45 minutes/session. Therapist monitoring: weekly phone-based contact with a coach.	(i) Verbal and visuospatial working memory	*Cognitive functioning*: (i) Abbreviated IQ of WASI (vocabulary, matrix reasoning) (ii) WISC IV (working memory, digit span forward, digit span backward, letter number sequencing, working memory index, spatial span forward, spatial span backward) (iii) CPT-II (omissions, hit reaction time) *Academic functioning*: (i) WJ-III (math fluency, reading fluency) *Everyday cognitive functioning*: (i) CPRS-3 (inattention, executive function) (ii) BRIEF (working memory scale, metacognitive index) *Other measures*: (i) Neuroimaging data	Improvement in (i) Working memory (spatial span backward) (ii) Attention (WISC-IV spatial span forward) (iii) Processing speed (CPT-II hit reaction time) (iv) Executive function (CPRS-3) (v) Functional magnetic resonance imaging revealed reduction in activation of left lateral prefrontal and bilateral medial frontal areas.

Eve et al., [64]	Pre/post-test design. Follow-up: at 12 months. Control group: no control group.	*N* = 7 children (5 at follow-up). Age: 10-16 years. Diagnosis: arterial ischemic stroke, at 4-10 years since injury. Inclusion criteria based on cognitive functioning: (i) IQ scores within 2 SDs of the mean, as measured by the WASI or WISC 4	Training: Cogmed Working Memory Training. Description: this program consists of exercises targeting verbal and visuospatial working memory. Training adaptation: the difficulty of exercises was automatically and in real time adjusted by the program, based on the child's performance. Setting: home. Duration: 25 sessions. Frequency: 5 sessions/week, about 30-40 minutes per day. Therapist monitoring: weekly phone-based contact with the coach.	(i) Verbal and visuospatial working memory	*Cognitive functioning*: (i) WMTB-C (digit recall, word list matching, word list recall and non-word list recall, block recall, listening recall, backward digit recall) (ii) Tea-Ch: (Sky Search, Score!, Score! Dual Task, Sky Search Dual Task, Walk/Do not Walk) *Academic functioning*: (i) WRAT-4 (mathematics)	Improvement in (i) Verbal working memory (not maintained at follow-up)

Phillips et al., [50]^∗∗^	Double-blind, randomized, placebo-controlledtrial. Pre/posttest design. Follow-up: at 3 months. Control group: control group performing non-adaptive tasks of a less demanding version of the Cogmed Working Memory Training.	*N* = 27 (E: 13, C: 14). Age: 8-15 years. Diagnosis: moderate to severe TBI, at >12 months since injury. Inclusion criteria based on cognitive functioning: (i) FSIQ > 80	Training: Cogmed Working Memory Training. Description: this program consists of exercises targeting verbal and visuospatial working memory. Training adaptation: the difficulty of exercises was automatically and in real time adjusted by the program, based on the child's performance. Setting: home. Duration: 25 sessions. Frequency: 5 sessions/week, about 30-40 minutes per day. Therapist monitoring: weekly phone-based contact with a coach.	(i) Visuospatial and verbal working memory	*Cognitive functioning:* (i) AWMA (digit recall, dot matrix) (ii) TEA-Ch (Sky Search Score, Sky Search DT, Creature Counting, Walk/Do not Walk) *Academic functioning:* (i) WIAT-II (word reading, reading comprehension, numerical operations)	Improvement in: (i) WM tasks (at post-trainingand follow-up) (ii) Reading comprehension (at post-training) (iii) Reading accuracy (at follow-up)

Sakzewski et al., [52]^∗∗^	Randomized controlled trial. Pre/posttest design. Control group: control group undergoing usual care program.	*N* = 58 (E: 29, C: 29). Age: 8-16 years. Diagnosis: stroke (*N* = 22; E: 10, C: 12), TBI (*N* = 19, E: 11, C: 8), nontraumatic ABI (*N* = 17; E: 8, C: 9), at least 12 months since injury.	Training: “Move it to improve it” (Mitii™). Description: this training is a multimodal web-based rehabilitation intervention to improve occupational performance, visual perception, and upper limb speed. Training adaptation: the therapist weekly adjusted the exercise complexity based on child's performance and feedback from the child and family. Setting: home. Duration: 20 weeks. Frequency: 6 days/week, 30 minutes/day. Therapist monitoring: weekly remote monitoring provided by a therapist for motivation and troubleshooting.	(i) Physical domain (i.e., upper limb and gross motor abilities) (ii) Visuospatial perception (visual discrimination, spatial relations, visual memory, form constancy, sequential memory, figure ground, and visual closure)	*Motor functioning*: (i) AMPS (processing, motor skills) (ii) Jebsen-Taylor Test of Hand Function (iii) COPM (performance, satisfaction) (iv) AHA (school kids version) *Cognitive functioning*: (i) TVPS (overall standard score, discrimination, memory, spatial relations, sequential memory, figure ground, and closure)	Improvement: negligible changes

Treble-Barna et al., [33]	Open-label pilot study. Pre/posttest design. Control group: healthy control group (*n* = 11) receiving the same treatment.	*N* = 24 (E: 13, C: 11). Age: 9-15 years. Diagnosis: mild to severe TBI, at least 1 year since injury. Inclusion criteria based on cognitive functioning: (i) Evidence of behavioral attention problems (Vanderbilt ADHD Diagnostic Parent Rating Scale, Attention Subscale: at least 4 items with a frequency score of 2 or 3)	Training: Attention Improvement and Management (AIM). Description: this training is a cognitive intervention aimed at improving attention and executive functions. The program combines computerized drill-based tasks and metacognitive strategy instruction. Training adaptation: the difficulty of exercises was weekly adjusted by a therapist. Setting: home. Duration: 12 weeks. Frequency: 2-4 sessions/week. Therapist monitoring: weekly face-to-face meetings between the child and the research clinician were scheduled to review home practice and adjust exercise difficulty. Weekly face-to-face meetings between the child and the research clinician were scheduled to review home practice and adjust exercise difficulty.	(i) Working memory (ii) Attention (inhibition, sustained, selective/focused attention) (iii) Executive functions (flexibility of thinking, inhibition, problem solving, planning, impulse control, concept formation, abstract thinking, and creativity in both verbal and spatial modalities)	*Cognitive functioning*: (i) TEA-Ch (the Score!, Walk/Do not Walk, Code Transmission, Sky Search) (ii) D-KEFS (Number-Letter Switching score from TMT, Inhibition/Switching score from CWIT, Total Achievement Score from the Tower Test) *Everyday cognitive functioning*: (i) BRIEF (BRI, GEC, MI) *Other measures*: (i) GAS	Improvement in (i) Sustained attention (ii) Parent-reportedEFs (iii) The majority of families also reported expected or more-than-expectedpersonalized goal attainment

Carlson-Green et al., [44]	Pre/posttest design. Follow-up: at 6 months. Control group: no control group.	*N* = 20. Age: 8-18 years. Diagnosis: BT, at least 1 year since surgery and/or adjuvant therapies. Inclusion criteria based on cognitive functioning: (i) Working memory deficits (T − score ≥ 75th percentile on the attention scale of CBCL or T − score ≥ 75th percentile on the parent-rated working memory scale of BRIEF or WMI (WISC 4) > 1 SD below the mean) (ii) FSIQ ≥ 70	Training: Cogmed Working Memory Training. Description: this program consists of exercises addressing verbal and visuospatial working memory. Training adaptation: the difficulty of exercises was automatically and in real time adjusted by the program, based on the child's performance. Setting: home. Duration: 35 sessions, in 8-12 weeks. Frequency: not reported. Therapist monitoring: intermittent emails from coaches.	(i) Visuospatial and verbal working memory	*Cognitive functioning*: (i) AWMA (at the baseline and immediately after training: digit recall, nonword recall, listening recall, backwards digit recall, dot matrix, mister X, mazes memory, and odd one out; at the baseline and at 6-month follow-up: word recall, digit recall, backwards digit recall, counting recall, dot matrix, mister X, and spatial recall) *Academic functioning*: (i) WJ-III (applied problems and passage, comprehension subtests) *Behavioral, social, and emotional functioning*: (i) CBCL (internalizing scale, externalizing scale, total problems scale) *Everyday cognitive functioning*: (i) BRIEF, at baseline and 6-month assessment *Social functioning*: (i) ABAS-II, at baseline and 6-month follow-up *Other measures*: (i) NPS: index of a child's exposure to neurocognitive risk factors during cancer treatment	Improvement in (i) Working memory at post training and at follow-up (i) Math achievement at follow-up (i) Executive functioning at follow-up

Conklin et al., [45] (extension of Conklin et al., [46])	Single-blind randomized controlled trial. Pre/posttest design. Follow-up: at 6 months. Control group: passive control group.	*N* = 68 (E: 34, 23 ALL +11 BT; C: 34, 24 ALL +10 BT). Age: 8-16 years. Diagnosis: ALL or BT, at least 1 year since surgery and/or adjuvant therapies, without recurrence. Inclusion criteria based on cognitive functioning: (i) Digit span, letter-number sequencing, or spatial span performance (WISC-IV) > 1 SD below the normative mean (ii) IQ > 70 (WASI)	Training: Cogmed Working Memory Training. Description: this program consists of exercises targeting verbal and visuospatial working memory. Training adaptation: the difficulty of exercises was automatically and in real time adjusted by the program, based on the child's performance. Setting: home. Duration: 25 sessions, in 5-9 weeks, 30-45 minutes/session. Frequency: not reported. Therapist monitoring: weekly phone-based contact with a coach.	(i) Verbal and visuospatial working memory	*Cognitive functioning*: (i) Abbreviated IQ of WASI (vocabulary+matrix reasoning) (ii) WISC IV (working memory, digit span forward, digit span backward, letter number sequencing, working memory index, spatial span forward, spatial span backward) (iii) CPT-II (omissions, hit reaction time) *Academic functioning*: (i) WJ-III (math fluency, reading fluency) *Everyday cognitive functioning*: (i) CPRS-3 (inattention, executive function) (ii) BRIEF (working memory, metacognitive index) *Other measures*: (i) Neuroimaging data	Improvement: (i) Working memory and processing speed were unchanged from posttest to 6 month follow-up, indicating maintenance of training improvement.

Piovesana et al., [51]^∗∗^	Randomized waitlist controlled trial. Control group: waiting-list control group.	*N* = 60 (E: 30, C: 30). Age: 8-16 years. Diagnosis: mild, moderate, or severe ABI, at least 12 months since injury.	Training: “Move it to improve it” (Mitii™). Description: Internet-based, multimodal program aimed at exercising cognitive, visual perceptual, and physical (i.e., upper limb and gross motor) function areas. Training adaptation: weekly remote monitoring of a therapist to control performance and update the program difficulty. Setting: home. Duration: 20 weeks. Frequency: 6 days/week for 30 minutes. Therapist monitoring: regular contact via phone or email with participants and families by the therapist to provide feedback and support.	(i) Cognitive functions (ii) Visual-perceptualfunctions (iii) Physical functions (i.e., upper limb and gross motor abilities)	*Cognitive functioning*: (i) WISC 4 (digit span backward, symbol search, coding) (ii) CTMT (trail 2, 3, 4, 5) (iii) D-KEFS: color naming, word reading, inhibition scores from CWI (iv) TOL (v) TEA-Ch (Sky Search, Sky Search Dt Score) *Everyday cognitive functioning*: (i) BRIEF (BRI, MI, GEC)	Improvement: none

Verhelst et al., [68]	Pre/posttest design. Feasibility study with preliminary data on efficacy. Follow-up: at 6 months. Control group: no control group.	*N* = 5. Age: 16-17 years. Diagnosis: TBI, at least 1 year, but no more than 5 years since injury.	Training: Brain Games Program. Description: entertaining games aimed at stimulating working memory/executive functioning and attention. Training adaptation: the difficulty of exercises was automatically and in real time adjusted by the program, based on the child's performance. Setting: home. Duration: 8 weeks. Frequency: 5 sessions/week. Therapist monitoring: none. Participants received a calendar and a training schedule to self-monitor their own adherence and performance.	(i) Attention (ii) Working memory (iii) Processing speed	*Cognitive functioning*: (i) CPT III (ii) Flanker task (III) WISC III: digit span-forward, digit-span backward, digit symbol coding (iv) CANTAB: spatial span-forward and backward, stockings, intra-extradimensionalset shift *Everyday cognitive functioning*: (i) BRIEF (BRI, MI, GEC)	Improvement: (i) Positive effect sizes were found for all measures, except the BRIEF (BRI).

Corti et al., [29]	Pre/posttest design. Feasibility study with preliminary data on efficacy. The main study on efficacy is a randomized controlled study, with a stepped-wedge design. Control group (in the main study): waiting-list group, performing the same cognitive training of the experimental group at a different time point.	*N* = 32. Age: 11-16 years. Diagnosis: 28 ABI (traumatic or nontraumatic) + 4 congenital brain damage, at least 1 year since injury.	Training: Lumosity Cognitive Training. Description: Lumosity Cognitive Training is a brain training consisting of different games, each targeting one of the following cognitive domains: speed of processing, attention, memory, cognitive flexibility, and problem solving. Five of the possible brain-training exercises of the program were chosen for this study. Training adaptation: the difficulty of exercises was automatically and in real time adjusted by the program, based on the child's performance. Setting: home. Duration: 8 weeks. Frequency: 20 minutes/day, 5 days/week. Therapist monitoring: weekly remote monitoring provided by a researcher for motivation and troubleshooting.	(i) Memory (ii) Attention (iii) Executive functioning (iv) Processing speed (v) Arithmetic operations	*Cognitive functioning*: (i) Lumosity Performance Index (LPI): average performance across the different games	Improvement: (i) Increase in mean LPI between the first day and the last day of the training (Cohen's *d* = 0.80)

Vander Linden et al., [67] (extension of Verhelst et al., [68])	Pre/posttest design. Control group: passive control group.	*N* = 32 (E: 16, C: 16). Age: 11-17 years (mean: 15.8 years). Diagnosis: moderate to severe TBI (mean age at injury: 13.4 years, mean time since injury: 2.4 years).	Training: Brain Games Program. Description: entertaining games aimed at stimulating working memory/executive functioning and attention. Training adaptation: the difficulty of exercises was automatically and in real time adjusted by the program, based on the child's performance. Setting: home. Duration: 8 weeks. Frequency: 40 minutes/session, 5 days/week. Therapist monitoring: none. Participants received a calendar and a training schedule to self-monitor their own adherence and performance.	(i) Memory (verbal memory, visuospatial memory, and working memory) (ii) Attention (selective attention, sustained attention, and inhibition) (iii) Processing speed (iv) Nonverbal learning (v) Problem-solving and planning	*Cognitive functioning*: (i) Digit span (forward and backward) of WISC IV (ii) Spatial span (forward and backward) of WISC IV (iii) Flanker task (conflict cost) (iv) CPT III (reaction time) (v) Digit Symbol Substitution Test (vi) Stockings of Cambridge *Everyday cognitive functioning*: (i) BRIEF (GEC)	Improvement in (i) Digit span forward, Flanker test, CPT III, digit symbol substitution, stockings of Cambridge, and GEC at posttraining and at 6-month follow-up (ii) Digit span backwards at 6-monthfollow-up (iii) Executive functions in daily living (BRIEF) for patients without diffuse-axonal-injuriesin deep brain nuclei

Note: ^∗∗^study included in the meta-analytic exploration. Inclusion criteria indicated in the table only refers to those reported in the studies with respect to cognitive functioning. Other inclusion criteria based on demographic factors were omitted. ABAS-II: Adaptive Behavior Assessment System, 2nd Edition; ABI: acquired brain injury; AHA: The Assisting Hand Assessment; AIS: arterial ischemic stroke; ALL: acute lymphoblastic leukemia; AMPS: Assessment of Motor and Process Skills; ANT: Amsterdamse Neuropsychologische Taken Programme; AWMA: Automated WM Assessment; BRI: Behavioral Regulation Index; BT: brain tumor; C: control group; CANTAB: Cambridge Automated Neuropsychological Test Battery; CBCL: Child Behavioral Checklist; COPM: Canadian Occupational Performance Measure; CPRS: Conners' Parent Rating Scale; CPT: Continuous Performance Test; CTMT: Comprehensive Trail Making Test; CWIT: Color-Word Interference Test; DKEFS: Delis Kaplan Executive System; DSM-IV: Diagnostic and Statistical manual, 4th edition; E: experimental group; EF: executive functions; FSIQ: Full Scale Intelligence Quotient; GAS: Goal Attainment Scale; GCS: Glasgow Coma Scale; GEC: Global Executive Composite; KABC-II: Kaufman Assessment Battery for Children, 2nd edition; GMFCS: Gross Motor Function Classification Scale; MI: Metacognitive Index; MVPT-3: Motor Free Test of Visual Perception, 3rd Edition; NPS: Neurological Predictor Scale; NEPSY II: Developmental NEuroPSYchological Assessment; PRI: Perceptual Reasoning Index; PSI: Processing Speed Index; SD: standard deviation; TEA-Ch: Test of Everyday Attention for Children; TMT: The Trail Making Test; TOVA: Test of Variables of Attention; TT: Tower Test; TVPS: Test of Visual Perceptual Skills; WAIS-III: Wechsler Intelligence Scale, 3rd edition; WASI: Wechsler Abbreviated Scale of Intelligence; WISC IV: Wechsler Intelligence Scale for Children, 4th edition; VCI: Verbal Comprehension Index; WIAT-II: Wechsler Individual Achievement Tests, 2nd Edition; WJ-III: Woodcock-Johnson 3rd Edition; WM: working memory; WMI: Working Memory Index; WMTB-C: Working Memory Test Battery for Children; WRAML2: Wide Range Assessment of Learning and Memory, 2nd Edition; WRAT-3: Wide Range Achievement Test, 3rd edition; WRAT-4: Wide Ranging Achievement Test, 4th edition.

**Table 2:** Overview of the studies on Behavioral Training Programs.

Study	Research design	Sample	Treatment characteristics	Treatment domain(s)	Outcome measures	Main findings
Wade et al., [72]	Pilot study. Pre/posttest design.	*N* = 6. Age: 5-16 years. Diagnosis: mild to severe TBI at more than 15 months since injury.	Training: OFPS. Setting: home. Duration: 6 months for the 8 core sessions +4 supplemental sessions. Therapist monitoring: weekly Skype sessions with a therapist.	(i) Everyday executive functions (ii) Behavioral, social, and emotional functioning	*Everyday executive functions*: (i) BRIEF (parent report): GEC *Behavioral, social, and emotional functioning*: (i) HCSBS (parent report): antisocial behavior subscale (ii) CDI (self-report)	Reduction of antisocial behaviors.

Wade et al., [55]^∗∗^	Randomized controlled study. Pre/posttest design. Control group: active control group, assigned to IRC.	*N* = 39 (E: 20, C: 19). Age: 5-16 years. Diagnosis: mild to severe TBI at 1-24 months since injury.	Training: OFPS. Setting: home. Duration: 6 months for the 8 core sessions +6 eventual supplemental sessions. Therapist monitoring: Skype sessions with a therapist every 1-2 weeks.	(i) Everyday executive functions (ii) Behavioral, social, and emotional functioning	*Behavioral, social, and emotional functioning*: (i) CBCL (parent report): externalizing, internalizing, and total problems (ii) HCSBS (parent report): peer total, self-management/compliance, total scale	Improvement in self-control and compliance with parents.

Wade et al., [22]	Randomized controlled study. Pre/posttest design. Control group: active control group, assigned to the same treatment program (TOPS), but without audio.	*N* = 9 (E: 5, C: 4). Age: 11-18 years. Diagnosis: mild to severe TBI at <24 months since injury.	Training: TOPS with audio. Setting: home. Duration: 6 months for the 10 core sessions +4 eventual supplemental sessions. Therapist monitoring: Skype sessions with a therapist every 1-2 weeks.	(i) Everyday executive functions (ii) Behavioral, social, and emotional functioning	*Everyday executive functions*: (i) BRIEF (parent report): GEC *Behavioral, social, and emotional functioning*: (i) CBCL (parent report): externalizing, internalizing, and total problems (ii) CDI (self-report)	Reduction of self-reported depression symptoms and of internalizing symptoms in children of both groups. Adolescent assigned to the audio condition showed a greater improvement in internalizing symptoms.

Wade et al., [23]^∗∗^	Randomized controlled study. Pre/posttest design. Control group: active control group, assigned to IRC.	*N* = 35 (E: 16; C: 19). Age: 11-18 years. Diagnosis: mild to severe TBI at <18 months since injury.	Training: TOPS. Setting: home. Duration: 6 months for the 10 core sessions +4 eventual supplemental sessions. Therapist monitoring: Skype sessions with a therapist every 1-2 weeks.	(i) Everyday executive functions (ii) Behavioral, social, and emotional functioning	*Everyday executive functions*: (i) BRIEF (parent and youth self-report): GEC, BRI, MI	Significant improvement in executive function behaviors in teens with severe TBI.

Wade et al., [58]^∗∗^	Randomized controlled study. Pre/posttest design. Control group: active control group, assigned to IRC.	*N* = 35 (E: 16, C: 19). Age: 11-18. Diagnosis: mild to severe TBI at 3-19 months since injury.	Training: TOPS. Setting: home. Duration: 6 months for the 10 core sessions +4 eventual supplemental sessions. Therapist monitoring: Skype sessions with a therapist every 1-2 weeks.	(i) Everyday executive functions (ii) Behavioral, social, and emotional functioning	*Behavioral, social, and emotional functioning*: (i) CBCL (parent and youth self-report): externalizing and internalizing problems	Reduction of parent-teen conflict. Reduction of internalizing symptoms among adolescents with severe TBI.

Kurowski et al., [53]^∗∗^	Randomized controlled study. Pre/posttest design. Control group: active control group, assigned to IRC.	*N* = 110 (E: 57, C: 63). Age: 12-17 years. Diagnosis: mild to severe TBI at 1-6 months since injury.	Training: CAPS. Setting: home. Duration: 6 months for the 10 core sessions +6 eventual supplemental sessions. Therapist monitoring: weekly Skype sessions with a therapist in months 1-3; be-weekly Skype sessions in months 4-6.	(i) Everyday executive functions (ii) Behavioral, social, and emotional functioning	*Everyday executive functions*: (i) BRIEF (parent report): GEC, BRI, MI	Improvement in executive function behaviors observed in older teens.

Kurowski et al., [69] (extension of Kurowski et al., [53])	Randomized controlled study. Pre/posttest design. Follow-up: at 12 and 18 months. Control group: active control group, assigned to IRC.	*N* = 131 (E: 65, C: 66). Age: 12-17 years. Diagnosis: mild to severe TBI at 1-6 months since injury.	Training: CAPS. Setting: home. Duration: 6 months, for the 10 core sessions +6 eventual supplemental sessions. Therapist monitoring: weekly Skype sessions with a therapist in months 1-3; be-weekly Skype sessions in months 4-6.	(i) Everyday executive functions (ii) Behavioral, social, and emotional functioning	*Everyday executive functions*: (i) BRIEF (parent report): GEC, BRI, MI	Improvement in executive function behaviors observed in older teens both at 12- and 18-month follow-up.

Wade et al., [56]^∗∗^	Randomized controlled study. Pre/posttest design. Control group: active control group, assigned to IRC.	*N* = 131 (E: 65, C: 66). Age: 12-17 years. Diagnosis: mild to severe TBI at 1-6 months since injury.	Training: CAPS. Setting: home. Duration: 6 months, for the 10 core sessions +6 eventual supplemental sessions. Therapist monitoring: weekly Skype sessions with a therapist in months 1-3; be-weekly Skype sessions in months 4-6.	(i) Everyday executive functions (ii) Behavioral, social, and emotional functioning	*Behavioral, social, and emotional functioning*: (i) CBCL (parent report): externalizing and internalizing problems+aggressive, attention, ADHD, and conduct scales	Improvement in externalizing symptoms in older teens.

Wade et al., [70] (extension of Kurowski et al., 2011, [53]; Wade et al., [56])	Randomized controlled study. Pre/posttest design. Follow-up: at 12 and 18 months. Control group: active control group, assigned to IRC.	*N* = 131 (E: 65, C: 66). Age: 12-17 years. Diagnosis: mild to severe TBI at <7 months since injury.	Training: CAPS. Setting: home. Duration: 6 months, for the 10 core sessions +6 eventual supplemental sessions. Therapist monitoring: weekly Skype sessions with a therapist in months 1-3; be-weekly Skype sessions in months 4-6.	(i) Everyday executive functions (ii) Behavioral, social, and emotional functioning	*Behavioral, social, and emotional functioning*: (i) CAFAS (parent report): total scale	Improvement in everyday functioning, especially in families of lower socioeconomic status.

Wade et al., [71] (extension of Wade et al., [56])	Randomized controlled study. Pre/posttest design. Follow-up: at 12 and 18 months. Control group: active control group, assigned to IRC.	*N* = 132 (E: 65, C: 67). Age: 12-17 years. Diagnosis: mild to severe TBI at 1-6 months since injury.	Training: CAPS. Setting: home. Duration: 6 months, for the 10 core sessions +6 eventual supplemental sessions. Therapist monitoring: weekly Skype sessions with a therapist in months 1-3; be-weekly Skype sessions in months 4-6.	(i) Everyday executive functions (ii) Behavioral, social, and emotional functioning	*Behavioral, social, and emotional functioning*: (i) CBCL (parent report): externalizing and internalizing problems	Improvement in behavior problems in older adolescents and those with pretreatment symptoms.

Tlustos et al., [54]^∗∗^	Randomized controlled study. Pre/posttest design. Control group: active control group, assigned to IRC.	*N* = 132 (E: 65, C: 67). Age: 11-18 years. Diagnosis: mild to severe TBI at 1-6 months since injury.	Training: CAPS. Setting: home. Duration: 6 months, for the 10 core sessions +6 eventual supplemental sessions. Therapist monitoring: weekly Skype sessions with a therapist in months 1-3; be-weekly Skype sessions in months 4-6.	(i) Everyday executive functions (ii) Behavioral, social, and emotional functioning	*Everyday executive functions*: (i) BRIEF (parent and youth self-report): GEC *Behavioral, social, and emotional functioning*: (i) HCSBS (parent report): social competence subscale (ii) CBCL (parent report): social competence subscale (iii) BERS 2 (parent report): social competence subscale	Improvement in social competence. Greater improvement in younger teens with moderate TBI.

Narad et al., [73]	Pilot study. Pre/posttest design. Control group: no control group.	*N* = 4. Age: 14-17 years. Diagnosis: mild to severe TBI during childhood (mean age at injury: 7.27 years, mean time since injury: 8.46 years).	Training: SPAN. Setting: home. Duration: 4 weeks. Therapist monitoring: weekly videoconferencing sessions via Skype with a trained college-student coach.	(i) Behavioral, social, and emotional functioning	*Behavioral, social, and emotional functioning*: (i) CBCL (parent and youth self-report): social competence, social problems and total problems scales	Improvement in social participation. Medium to large effect sizes were found for adolescent self-reported measures. Negligible effects were observed for parent-reported measures.

Wade et al., [48]	Pilot study. Pre/posttest design. Control group: no control group.	*N* = 15. Age: 14-22 years. Diagnosis: 9 TBI (moderate or severe); 6 BT (at least 2 years out from treatment completion or from diagnosis if treatment was not indicated).	Training: SPAN. Setting: home. Duration: 10 weeks. Monitoring: weekly videoconferencing sessions via Skype with a coach for 30-60 minutes.	(i) Behavioral, social, and emotional functioning	*Behavioral, social, and emotional functioning*: (i) CBCL (parent and youth self-report): internalizing, externalizing, and total problems scales+social problems and social competence scales	Improvement in parent reported frequency of social participation and total, internalizing, and externalizing problems; improvement in teen-reported confidence ability to participate and develop social participation goals and plans.

Wade et al., [57]^∗∗^	Randomized controlled study. Pre/posttest design. Control group: two versions of the TOPS program (TOPS-F and TOPS-TO) were compared with an active control group, assigned to IRC.	*N* = E1 (TOPS-TO): 51; E2 (TOPS-F): 49, C: 52. Age: 11-18 years. Diagnosis: mild to severe TBI at <18 months since injury.	Training: TOPS-TO or TOPS-F. Setting: home. 6 months for the 10 core sessions +4 eventual supplemental sessions. Monitoring: Skype sessions with a therapist every 1-2 weeks.	(i) Everyday executive functions (ii) Behavioral functioning	*Behavioral, social, and emotional functioning*: (i) CBCL (parent and youth self-report): externalizing problems *Everyday executive functions:* (i) BRIEF (parent and youth self-report): GEC	Improvement in executive functioning in the TOPS-F group, as compared to the TOPS-TO group. Differences between the TOPS-F and IRC groups approached significance. Maternal education and parental stress levels moderated improvements.

Note: ^∗∗^study included in the meta-analytic exploration. Outcomes reported in the table refer only to children. ADHD: attention deficit and hyperactivity disorder; BERS 2: Behavioral and Emotional Rating Scale 2; BRI: Behavioral Regulation Index; BRIEF: Behavior Rating Inventory of Executive Function; BT: brain tumor; C: control group; CAFAS: Child and Adolescent Functional Assessment Scale; CAPS: Counselor-Assisted Problem Solving; CBCL: Child Behavior Checklist; CDI: Children's Depression Inventory; E: experimental group; GEC: Global Executive Composite; HCSBS: Home and Community Behavior Scale; IRC: Internet Resource Comparison; MI: Metacognition Index; OFPS: Online Family Problem-Solving; SES: socioeconomic status; SPAN: Social Participation and Navigation; TBI: traumatic brain injury; TOPS: Teen Online Problem-Solving; TOPS-F: Teen Online Problem-Solving-Family; TOPS-TO: Teen Online Problem-Solving-Teen Only; WEQ: Website Evaluation Questionnaire.

**Table 3:** Risk of bias summary.

Study	Selection bias		Performance bias	Detection bias	Attrition bias	Reporting bias
	Random sequence	Allocation	Blinding of participants and personnel	Blinding of assessors	Incomplete outcome data	Selective reporting
*Cognitive Training Programs*						
Bangirana et al., [36]^∗∗^	+	—	—	—	+	?
Bangirana et al., [35]^∗∗^	+	?	—	—	+	?
Carlson-Green et al., [44]	/	/	/	—	+	?
Conklin et al., [46]^∗∗^	+	?	?	+	—	?
Conklin et al., [45]	+	?	?	+	+	?
Corti et al., [29]	+	—	+	+	+	?
de Kloet, et al., [34]	/	/	/	?	+	?
Eve, et al., [64]	/	/	/	?	—	?
Hardy et al., [65]	/	/	/	?	—	?
Hardy et al., [47]^∗∗^	+	?	+	+	—	?
Kesler et al., [21]	/	/	/	+	+	?
Phillips et al., [50]^∗∗^	+	+	+	+	+	?
Piovesana et al., [51]^∗∗^	+	+	+	?	—	?
Sakzewski et al., [52]^∗∗^	+	+	—	+	+	?
Sohlberg et al., [66]	/	/	/	—	—	?
Treble-Barna et al., [33]	/	/	/	—	+	?
Vander Linden et al., [14]	/	/	?	+	+	?
Verhelst et al., [68]	/	/	/	?	+	?

*Behavioral Training Programs*						
Kurowski et al., [53]^∗∗^	+	+	—	+	?	?
Kurowski et al., [69]	+	+	—	+	?	?
Narad et al., [73]	/	/	/	?	+	?
Tlustos et al., [54]^∗∗^	?	?	—	+	?	?
Wade et al., [72]	/	/	/	?	+	?
Wade et al., [55]^∗∗^	+	—	—	—	—	?
Wade et al., [22]	+	—	—	—	+	—
Wade et al., [23]^∗∗^	+	—	—	—	—	?
Wade et al., [58]^∗∗^	+	—	—	—	—	—
Wade et al., [56]^∗∗^	+	+	—	+	?	?
Wade et al., [70]	+	+	—	+	?	?
Wade et al., [71]	+	+	—	+	?	?
Wade et al., [48]	/	/	/	?	?	?
Wade et al., [57] ^∗∗^	+	?	—	?	?	?

Symbol legend: +: low risk of bias; -: high risk of bias; /: not applicable (e.g., allocation to treatments in a study with no control group); ?: unclear risk of bias. Asterisks ^∗∗^ mark studies included in the meta-analytic exploration.
